# E-SNPs&GO: embedding of protein sequence and function improves the annotation of human pathogenic variants

**DOI:** 10.1093/bioinformatics/btac678

**Published:** 2022-10-13

**Authors:** Matteo Manfredi, Castrense Savojardo, Pier Luigi Martelli, Rita Casadio

**Affiliations:** Biocomputing Group, Department of Pharmacy and Biotechnology, University of Bologna, Bologna 40126, Italy; Biocomputing Group, Department of Pharmacy and Biotechnology, University of Bologna, Bologna 40126, Italy; Biocomputing Group, Department of Pharmacy and Biotechnology, University of Bologna, Bologna 40126, Italy; Biocomputing Group, Department of Pharmacy and Biotechnology, University of Bologna, Bologna 40126, Italy

## Abstract

**Motivation:**

The advent of massive DNA sequencing technologies is producing a huge number of human single-nucleotide polymorphisms occurring in protein-coding regions and possibly changing their sequences. Discriminating harmful protein variations from neutral ones is one of the crucial challenges in precision medicine. Computational tools based on artificial intelligence provide models for protein sequence encoding, bypassing database searches for evolutionary information. We leverage the new encoding schemes for an efficient annotation of protein variants.

**Results:**

E-SNPs&GO is a novel method that, given an input protein sequence and a single amino acid variation, can predict whether the variation is related to diseases or not. The proposed method adopts an input encoding completely based on protein language models and embedding techniques, specifically devised to encode protein sequences and GO functional annotations. We trained our model on a newly generated dataset of 101 146 human protein single amino acid variants in 13 661 proteins, derived from public resources. When tested on a blind set comprising 10 266 variants, our method well compares to recent approaches released in literature for the same task, reaching a Matthews Correlation Coefficient score of 0.72. We propose E-SNPs&GO as a suitable, efficient and accurate large-scale annotator of protein variant datasets.

**Availability and implementation:**

The method is available as a webserver at https://esnpsandgo.biocomp.unibo.it. Datasets and predictions are available at https://esnpsandgo.biocomp.unibo.it/datasets.

**Supplementary information:**

Supplementary data are available at *Bioinformatics* online.

## 1 Introduction

Single-nucleotide polymorphisms (SNPs) are major sources of human evolution. In many cases, these variations can be directly associated with the onset of genetic diseases. Specifically, SNPs occurring in protein-coding regions often lead to observable changes in the protein residue sequence. Single amino acid variations (SAVs) may have an impact at different levels, hampering protein structure, function, stability, localization and interaction with other proteins and/or nucleotides, hence setting the basis for the onset of pathologic conditions (Lappalainen and MacArthur, 2021; Vihinen, 2021 and references therein).

Public databases, such as HUMSAVAR (The UniProt Consortium, 2021) and ClinVar (Landrum *et al.*, 2018), store a compendium of known SAVs and provide, when available, information about the variant clinical significance. However, clear associations to diseases are still unknown for many SAVs, which substantially remain of Uncertain Significance (US). Therefore, SAV annotation is an issue, and effective computational tools are needed to provide large-scale annotation of uncharacterized human variation data.

In the past years, several computational approaches have been implemented, with the aim of annotating whether a protein variation is or not disease associated (Adzhubei *et al.*, 2010; Calabrese *et al.*, 2009; Carter *et al.*, 2013; Choi *et al.*, 2012; Jagadeesh *et al.*, 2016; Li *et al.*, 2009; Ng and Henikoff, 2001; Niroula *et al.*, 2015; Pejaver *et al.*, 2020; Raimondi *et al.*, 2017; Schwarz *et al.*, 2010; Yang *et al.*, 2022). Methods like SIFT (Ng and Henikoff, 2001) or PROVEAN (Choi *et al.*, 2012) are based on the conservation analysis in multiple sequence alignments. More complex approaches stand on different types of machine-learning frameworks. These include neural networks (Pejaver *et al.*, 2020), random forests (Carter *et al.*, 2013; Li *et al.*, 2009; Niroula *et al.*, 2015; Raimondi *et al.*, 2017), gradient tree boosting (Jagadeesh *et al.*, 2016; Yang *et al.*, 2022), support vector machines (SVMs) (Calabrese *et al.*, 2009) and naive Bayes classifiers (Adzhubei *et al.*, 2010; Schwarz *et al.*, 2010). Each method is trained/tested on different datasets of SAVs, either extracted directly from public resources like HUMSAVAR (The UniProt Consortium, 2021) and/or ClinVar (Landrum *et al.*, 2018), or taking advantage of pre-compiled datasets of variations, like VariBench (Nair and Vihinen, 2013). Different types of descriptors extract salient features of the protein sequence and/or the local sequence context surrounding the variant position, including physicochemical properties, sequence profiles, conservation scores, predicted structural motifs and functional annotations. SNPs&GO (Calabrese *et al.*, 2009) firstly recognized the importance of functional annotations for the prediction of variant pathogenicity and introduced the LGO feature, a score of association between Gene Ontology (GO) (Ashburner *et al.*, 2000) annotations and the variant pathogenicity. The incorporation of the LGO feature significantly improved the prediction performance of SNPs&GO (Calabrese *et al.*, 2009).

Recent developments in the field of deep learning focus on the definition of new ways of representing protein sequences. Large-scale protein language models (PLMs) are inspired and derived from the natural language processing (NLP) field (Ofer *et al*, 2021). They learn numerical vector representations of protein sequences, containing important features that are reflected in the evolutionary conservation and in the sequence syntax (Bepler and Berger, 2021). These numerical vectors are then adopted to encode protein sequence and/or individual residues in place of canonical, hand-crafted features, such as physicochemical properties or evolutionary information. These distributed protein representations emerge from the application of learning models trained on large databases of sequence data (Bepler and Berger, 2021; Ofer *et al.*, 2021).

Successful PLMs are routinely trained on databases composed of hundreds of millions of unique sequences with hundreds of billions of residues. Training is computationally demanding, routinely requiring weeks or months of computations on high-performance Tensor Processing Units (TPUs) and/or Graphical Processing Units (GPUs) (Elnaggar *et al.*, 2021; Rives *et al.*, 2021). However, the advantage is that most of the computational cost is concentrated on the training phase, and once models are trained they can be adopted to embed new sequences with limited resources in terms of time, memory and computational power.

Embeddings obtained with language models have been recently employed for many different applications with great success, including the prediction of protein function and localization (Littmann *et al.*, 2021; Stärk *et al.*, 2021; Teufel *et al.* 2022), of protein contact maps (Singh *et al.*, 2022) and binding sites (Mahbub and Bayzid, 2022).

Several pre-trained language models currently exist in the literature (Alley *et al.*, 2019; Asgari and Mofrad, 2015; Elnaggar *et al.*, 2021; Heinzinger *et al.*, 2019; Rives *et al.*, 2021; Strodthoff *et al.*, 2020), mainly differing in their specific architectures [autoregressive, bidirectional, masked; see for review Bepler and Berger (2021)] and in the datasets adopted for training.

Not limited to the encoding of protein sequence data, embedding techniques are also applied to model the relationships existing within more complex structures, such as graphs, networks, or biological ontologies (Edera *et al.*, 2022; Grover and Leskovec, 2016; Kandathil *et al.*, 2022; Perozzi *et al.*, 2014; Zhong *et al.*, 2019).

In this article, we attempt to fully exploit the power of language models and embeddings for the prediction of variant pathogenicity from the human protein sequence. On the methodological side, two major contributions can be highlighted. Firstly, we adopt two different and complementary embedding procedures, ProtT5 (Elnaggar *et al.*, 2021) and ESM-1v (Meier *et al.*, 2021), to directly encode an input variation without introducing any hand-crafted feature as previously done. Secondly, leveraging the idea introduced in SNPs&GO (Calabrese *et al.*, 2009), we explore a new way of encoding functional annotations by adopting a model called Anc2Vec (Edera *et al.*, 2022), specifically designed for the embedding of GO terms (Ashburner *et al.*, 2000).

We trained an SVM using the above input encoding on a newly generated dataset of 101 146 human disease-related and benign variations obtained from the rational merging of data deposited in two databases, HUMSAVAR (The UniProt Consortium, 2021) and ClinVar (Landrum *et al.*, 2018). The method is tested on an independent, non-redundant blind set comprising 10 266 variations, adopting stringent homology reduction and evaluation procedures. Results obtained in a comparative benchmark and including one of the most recent and effective methods (Pejaver *et al.*, 2020), demonstrate that our model performs at the level or even better than the state-of-the-art (when available for comparison) reaching a Matthews Correlation Coefficient (MCC) of 0.72. Based on an input encoding derived solely from embedding models, our method is fast: this makes it suitable for large-scale annotation of human pathogenic variants.

We release our tool as a webserver at https://esnpsandgo.biocomp.unibo.it.

## 2 Materials and methods

### 2.1 Dataset

We obtained the dataset of SAVs by merging information extracted from two resources: HUMSAVAR (accessed on August 4, 2021), listing all missense variants annotated in human UniProt/SwissProt (The UniProt Consortium, 2021) entries, and ClinVar (accessed on March 29, 2021), the NCBI resource of relationships among human variations and disease phenotypes (Landrum *et al.*, 2018).

Both databases classify the effect of SAVs into different classes: Pathogenic or Likely Pathogenic (P/LP), Benign or Likely Benign (B/LB) and of US. We retained only P/LP SAVs clearly associated with the diseases catalogued in OMIM (Amberger *et al.*, 2019) or in MONDO (Shefchek *et al.*, 2020). We collected also all the B/LB variations and excluded SAVs labelled as US, somatic, or with contrasting annotations of the effect.

Overall, the dataset consists of 13 661 protein sequences endowed with 111 412 SAVs, including 43 895 P/LP SAVs in 3603 proteins and 67 517 B/LB SAVs in 13 229 proteins (Table 1, last row).

**Table 1.** The dataset of SAVs adopted in this study

Dataset	No. of pathogenic SAVs	No. of neutral SAVs	No. of proteins
Training set	39 812	61 334	12 347
Blind test set	4083	6183	1314
Total	43 895	67 517	13 661

For all proteins in the dataset, we extracted GO (Ashburner *et al.*, 2000) annotations from the corresponding entry in UniProt. Overall, our dataset is annotated with 17 076 GO terms, including 11 476 Biological Process (BP), 3955 Molecular Function (MF) and 1645 Cellular Component (CC). The complete dataset is available at https://esnpsandgo.biocomp.unibo.it/datasets.

#### 2.1.1 Cross-validation procedure and generation of the blind test set

To avoid biases between training and testing sets, we adopted a stringent clustering procedure to generate cross-validation sets. Firstly, we clustered protein sequences with the MMseqs2 program (Steinegger and Söding, 2017), by constraining a minimum sequence identity of 25% over a pairwise alignment coverage of at least 40%. We used a connected component clustering strategy so that if two proteins are clustered with a third one, they both end up in the same set. In this way, we limit sequence redundancy between training and testing sets, enabling a fair evaluation of the results. We selected 10% of the data to construct the blind test set for assessing the generalization performance of our approach and for benchmarking it with other popular methods available. The remaining 90% of the dataset was further split into 10 equally distributed subsets that were used in a 10-fold cross-validation procedure for optimizing the input encoding and for fixing the model hyperparameters. We also tried a 20–80% split (20% of the data for the blind test set and 80% for training with the 10-fold cross-validation procedure) and obtained a very similar performance. For this reason, we list results corresponding to the 10% blind test. When performing cross-validation, we took care of preserving the balancing of positive and negative examples in each subset (Supplementary Table S1).

It is worth noticing that the blind test can share similarity with proteins included in the training sets of the other benchmarked methods.

### 2.2 General overview of the approach

**Fig. 1.**
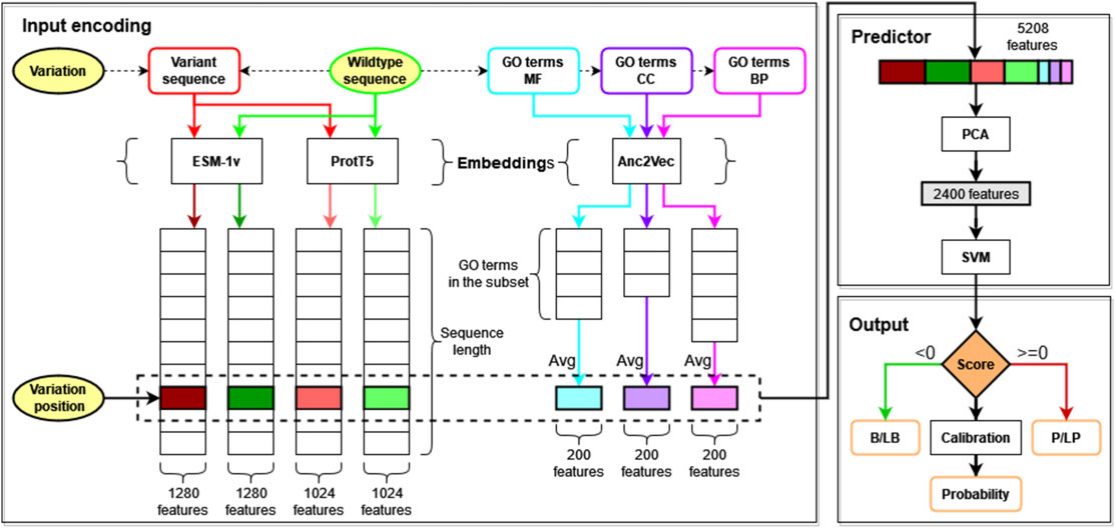
General overview of the architecture of E-SNPs&GO. Inputs (wild-type sequence, variation and variation position) are in yellow. The architecture includes three major blocks: an Input encoding, a Predictor and an Output. During the Input encoding, three embedding models are adopted to generate vector representations. The wild-type sequence (green) and the variant sequence (red) are modelled with ESM-1v (Meier *et al.*, 2021) and ProtT5 (Elnaggar *et al.*, 2021). The GO functional annotations (blue MF, purple CC and pink BP) are modelled with Anc2Vec (Edera *et al.*, 2022). The vectors within the dashed box (marked with different colors), representing the variation position and the averaged (Avg) GO terms of the wild-type sequence, are then concatenated together to obtain a final representation consisting of 1280×2 + 1024×2 + 200×3 = 5208 features. This vector is fed to the Predictor, which includes a PCA to reduce the input dimensionality (from 5208 to 2400) and a SVM providing as a final output a binary classification into B/LB (negative class, Score <0) or P/LP (positive class, Score ≥0). We apply an Isotonic Regression (Calibration) to obtain a calibrated probability (A color version of this figure appears in the online version of this article.)

Figure 1 depicts the architecture of E-SNPs&GO, including three major blocks: an Input encoding, a Predictor and an Output. The input consists of a human protein sequence and a SAV occurring at a specific position along the sequence. In the input encoding phase, the sequence and its variant are embedded with two different procedures, ESM-1v (Meier *et al.*, 2021) and ProtT5 (Elnaggar *et al.*, 2021), generating for each sequence 1280 and 1024 features, respectively. In order to embed the functional protein annotation of the wild-type protein, we adopt Anc2Vec (Edera *et al.*, 2022), computing three sets of 200 features corresponding to the different subontologies.

In the predictor, the vector representation generated in the input encoding is then processed using a principal component analysis (PCA), which reduces the dimensionality of the input from 5208 features to 2400. The output feeds a SVM classifier performing the final labelling as Pathogenic (P/LP) or Benign (B/LB). A given input variant is predicted as pathogenic when the SVM output score ≥0, benign otherwise. A final calibration step allows to convert scores into probabilities for a variant to be pathogenic. Details of the methods included in E-SNPs&GO, are listed in the following sections.

### 2.3 Input encoding: embeddings of protein sequence, its variant and GO terms

#### 2.3.1 Transformers for embedding of protein sequences and their variants

Several prominent language models and corresponding embedding generation schemes in NLP are available, and some of these have been adapted to protein sequences to perform specific prediction tasks (Bepler and Berger, 2021). Large-scale PLMs aim at learning a numerical vector representation that allows reconstructing the input sequence.

Among PLMs, transformer-based models (Vaswani *et al.*, 2017) aim to solve the problem of efficiently capturing long-distance interactions in the sequence. Transformers are architectures that include a self-attention mechanism to extract the context information from the whole sequence (Vaswani *et al.*, 2017). In general, a transformer language model builds on top of an encoder–decoder architecture. However, the different transformer-based PLMs only utilize either the encoder or the decoder part. In this respect, transformer-based PLMs can be classified in three different categories: (i) encoder-only models use only the encoder part of the transformer accessing the whole input sequence and are trained to reconstruct a somewhat corrupted version of the input (e.g. masking random positions along the sequence); (ii) decoder-only models (also called autoregressive models) use only the decoder part accessing, at each position, all the residues placed before the current one in the sequence and are usually trained to predict the next residue in the sequence; (iii) sequence-to-sequence models use both the encoder and the decoder and are trained to reconstruct a masked input sequence (Vaswani *et al.*, 2017).

The learned representation captures important features of the proteins, including physicochemical, structural, functional and evolutionary features (Bepler and Berger, 2021; Ofer *et al.*, 2021). By transfer learning, the embedded schemes are provided as input to Predictor block (Fig. 1).

In this article, we adopt two different protein embedding schemes, based on two different transformers models: ESM-1v (Meier *et al.*, 2021), an encoder-only model, and ProtT5 (Elnaggar *et al.*, 2021), a sequence-to-sequence model. The major difference stands in the volume of the sequence datasets used for generating the embedding schemes and in the adoption of different training procedures. ESM-1v was trained on a single run using a dataset of 98 million unique sequences extracted from UniRef90 (Suzek *et al.*, 2015). ESM-1v releases five models generated by training with five different random seeds (Meier *et al.*, 2021). Apparently, only a small difference in performance is obtained when the ensemble is compared to a single model (Meier *et al.*, 2021). Therefore, to reduce the computational cost, we adopted only one model (the first one). ProtT5 (version XL U50) was trained using a two-step procedure: in a first pass, training was performed using the large BFD database (Steinegger *et al.*, 2019; Steinegger and Söding, 2018), comprising the whole UniProt as well as protein sequences translated from multiple metagenomic sequencing projects, and consisting of about 2.1 billion unique sequences. In the second pass, a fine-tuning of the model was obtained using a smaller database derived from UniRef50 (Suzek *et al.*, 2015) and including 45 million unique sequences.

#### 2.3.2 Embedding of biological ontologies

The concept of embedding can be generalized to any kind of data with different underlying structures, such as graphs or networks (Grover and Leskovec, 2016; Perozzi *et al.*, 2014). In particular, several embedding models have been defined to provide a numerical representation of nodes in ontologies (Chen *et al.*, 2021; Zhong *et al.*, 2019). Here, we adopt Anc2Vec (Edera *et al.*, 2022), a method that learns a vector representation for GO terms, by preserving ancestor relationships.

Because the embedding is not context-dependent, we precompute the vector representation for each possible GO term.

### 2.4 Predictor

#### 2.4.1 Predictor input

For encoding variations, we firstly perform a full-sequence generation of embeddings using both the ESM-1v (Meier *et al.*, 2021) and the ProtT5 XL U50 (Elnaggar *et al.*, 2021) models. Given a protein sequence with *L* residues, this provides protein encodings of dimensions *L*×1280 and *L*×1024, respectively. Sequence embeddings are carried out independently on both the wild-type and the variant sequence.

For a variation at position *i* in a protein sequence, we compute a vector of 4608 features, including:

- 1280 features corresponding to ESM-1v embedding in position *i* of the variated sequence.
- 1280 features corresponding to ESM-1v embedding in position *i* of the wild-type sequence.
- 1024 features corresponding to ProtT5 (version XL U50) embedding in position *i* of the variated sequence.
- 1024 features corresponding to ProtT5 (version XL U50) embedding in position *i* of the wild-type sequence.

The ESM-1v embedding model constrains the maximal protein length (*L*) to 1024 residues. For this reason, variations occurring on longer sequences were encoded using a 201 long sequence window centred on the variant position.

After this step, we extract all the GO terms annotated in the UniProt entry of the wild-type protein carrying the variation. Potential term redundancy is removed by retaining only leaf terms. Terms from the three different GO sub-ontologies (MF, CC and BP) are processed independently. Each annotated GO term is then embedded as a vector of 200 features using the Anc2Vec model (Edera *et al.*, 2022). To obtain a single vector representation independent of the number of terms of a given protein, we average all the vector encodings (Fig. 1). Three final average vectors, one for each GO sub-ontology, are concatenated obtaining a protein function encoding of 600 components.

The final variation encoding comprises 5208 features, obtained by merging the local positional embedding (4608 features from ESM-1V + ProtT5 XL U50) described above and the Anc2Vec functional encoding (600 features). Eventually, we encode the different embeddings separately (see Section 3 and Table 2).

**Table 2.** Performance of different embedding schemes

Input encoding	*Q*_2_ (%)	Precision (%)	Recall (%)	*F*1-score (%)	ROC-AUC (%)	MCC
ESM-1v	82.4 (±1.5)	80.4 (±2.6)	77.0 (±2.8)	78.6 (±1.9)	81.6 (±1.5)	0.64 (±0.03)
ESM-1v+GO	83.3 (±1.4)	81.7 (±2.5)	78.1 (±2.7)	79.8 (±1.8)	82.6 (±1.4)	0.66 (±0.03)
ProtT5	83.0 (±1.3)	79.8 (±1.9)	80.0 (±2.8)	79.9 (±1.7)	82.6 (±1.4)	0.65 (±0.03)
ProtT5+GO	83.7 (±1.1)	81.8 (±1.9)	79.2 (±2.5)	80.5 (±1.5)	83.1 (±1.3)	0.67 (±0.02)
ESM-1v+ProtT5	83.6 (±1.4)	81.8 (±2.3)	78.6 (±2.9)	80.1 (±1.8)	82.9 (±1.5)	0.66 (±0.03)
ESM-1v+ProtT5+GO(-PCA)	83.1 (±0.8)	81.0 (±1.4)	78.0 (±1.5)	79.4 (±1.1)	82.8 (±0.8)	0.66 (±0.02)
ESM-1v+ProtT5+GO(+PCA)	85.1 (±0.9)	82.4 (±1.7)	79.1 (±1.7)	80.7 (±1.1)	84.1 (±0.9)	0.69 (±0.02)

*Note*: We adopted a 10-fold cross-validation on a training set comprising 101 146 human variations (Table 1) for testing the effect of different input encodings on the performances of the method. Standard deviation (between brackets) is computed over the 10 cross-validation sets and scoring indexes (defined in Section 2.6) are average values.

ESM-1v (2×1280 = 2560 features).

ESM-1v + GO (2×1280 + 3×200 = 3160 features).

ProtT5 (2×1024 = 2048 features).

ProtT5 + GO (2×1024 + 3×200 = 2648 features).

ESM-1v + ProtT5 (2×1280 + 2×1024 = 4608 features).

ESM-1v + ProtT5 + GO (−PCA) (2×1280 + 2×1024 + 3×200 = 5208 features), no PCA used.

ESM-1v + ProtT5 + GO (+PCA) (2×1280 + 2×1024 + 3×200 = 5208 features), PCA used to reduce dimensionality.

#### 2.4.2 Model selection and implementation

The predictor includes two cascading components (Fig. 1): a PCA for reducing the dimensionality of the input features and a binary SVM with a Radial Basis Function (RBF) kernel, which performs the variant classification into pathogenic or not. We optimized the hyperparameters of both methods (such as the number of components of PCA, the SVM cost parameter C and the gamma coefficient of the RBF kernel) with a grid search procedure. A complete list of hyperparameters tested and their optimal values are available in Supplementary Table S2.

It is worth clarifying that, during both cross-validation and blind testing, the execution of the PCA step is always computed on the training set and then applied for projecting vectors of the testing set in the reduced space.

All methods are implemented in Python3 using the scikit-learn library (Pedregosa *et al.*, 2011). ESM-1v and ProtT5 embeddings are computed with the bio-embeddings package (Dallago *et al.*, 2021).

The complete machine-learning workflow is compliant with the DOME recommendation checklist (Walsh *et al.*, 2021), as reported in Supplementary Table S3.

### 2.5 Output

The SVM adopted for classification computes a decision function that represents the distance of the point mapping the input from the discrimination boundary. We use this value to estimate the reliability of the prediction, in terms of the probability of the input variation to be pathogenic (Fig. 1).

In a perfectly calibrated method, when a set of predictions scored with probability *P* is tested on real data, we expect that the fraction of true positives is exactly *P*. In this work, we adopt a procedure previously described (Benevenuta *et al.*, 2021) to obtain a calibrated probability that we provide in output alongside the predicted class. In particular, we fit an Isotonic Regression (Niculescu-Mizil and Caruana, 2005) in cross-validation and we use it to obtain a probability score on the blind test. Supplementary Figure S1 shows that E-SNPs&GO output probabilities are very close to being perfectly calibrated, more than other popular methods.

Keeping as a reference the probability of being P/PL, the probability score (*P*_P/PL_) gives an integer Reliability Index from 0 (random prediction) to 10 (certain prediction) using the formula:

(1) $$\mathrm{RI}=\mathrm{round}\;\left(20\times \left|P_{P/LP}-0.5\right|\right).$$

### 2.6 Scoring indexes

We assess the performance with the following scores. P/LP variations are assumed to be the positive class, B/LB variations are the negative class. In what follows, TP, TN, FP and FN are true positive, true negative, false positive and false negative predictions, respectively.

We compute the following scoring measures:

- Accuracy (*Q*_2_):
  (2) $$Q_{2}=\frac{\mathrm{TP}+\mathrm{TN}}{\mathrm{TP}+\mathrm{TN}+\mathrm{FP}+\mathrm{FN}}.$$
- Precision:
  (3) $$\mathrm{Precision}=\frac{\mathrm{TP}}{\mathrm{TP}+\mathrm{FP}}.$$
- Recall:
  (4) $$\mathrm{Recall}=\;\frac{\mathrm{TP}}{\mathrm{TP}+\mathrm{FN}}.$$
- *F*1-score, the harmonic mean of precision and recall:
  (5) $$F1=\frac{2\times \mathrm{Precision}\times \mathrm{Recall}}{\mathrm{Precision}+\mathrm{Recall}}.$$
- Area under the receiver operating characteristic curve (ROC-AUC).
- MCC:
  (6) $$\mathrm{MCC}=\frac{\mathrm{TP}\times \mathrm{TN}-\mathrm{FP}\times \mathrm{FN}}{\sqrt{\left(\mathrm{TP}+\mathrm{FP})\times (\mathrm{TP}+\mathrm{FN})\times (\mathrm{TN}+\mathrm{FP})\times (\mathrm{TN}+\mathrm{FN}\right)}}.$$

## 3 Results

### 3.1 Assessing the contribution of different input encodings

To select the optimal input encoding, we performed different experiments to test various combinations of input features. To this aim, we trained in cross-validation several independent SVM+PCA models using different input features and using the MCC to score and select the optimal model.

GO terms provide global protein information. Their embedding does not consider the specific variant position. If the prediction is run considering only averaged embedded GO terms vector (Fig. 1), the predictor performance is very low (MCC = 0.27, data not shown). Different input encodings, corresponding to different predictors, perform differently (Table 2). The inclusion of GO embeddings in the final input is always beneficial, improving MCC by 2 or 3 percentage points in all cases (compare ESM-1v, ProtT5 and ESM-1v+ProtT5 with or without GO, respectively in Table 2). Considering the two protein sequence embeddings, ProtT5 outperforms ESM-1v both with and without the additional GO information. Most notably, the model trained on data from ProtT5 alone is the most balanced, reaching equal precision and recall. Finally, the concatenation of both sequence encodings and the GO embedding provides the best performance (MCC = 0.69), leading to an increase in precision without a corresponding decrease in recall.

Based on these results, we select the model trained with ESM-1v+ProtT5+GO as the optimal one.

### 3.2 Benchmark on the blind test set

We test our method adopting both a 10-fold cross-validation procedure and an independent blind test set constructed to be non-redundant with respect to the training dataset (see Section 2.1). Table 3 lists the results. E-SNPs&GO obtains similar results in cross-validation and blind test, making it very robust to generalization. Concerning individual indexes, our method seems to be slightly more precise than sensitive (compare Precision and Recall).

**Table 3.** Benchmark of our and other top scoring methods available in literature

Input encoding		*Q*_2_ (%)	Precision (%)	Recall (%)	*F*1-score (%)	ROC-AUC (%)	MCC
E-SNPs&GO^a^	Cross-validation	85.1 (±0.9)	82.4 (±1.7)	79.1 (±1.7)	80.7 (±1.1)	84.1 (±0.9)	0.69 (±0.018)
E-SNPs&GO^a^	Blind test set	86.8	85.7	80.1	82.8	85.6	0.72
SNPs&GO^a^	Blind test set	79.8	84.8	63.2	72.4	77.5	0.58
MutPred2.0^b^	Blind test set	85.6	78.6	87.7	82.9	85.9	0.71
PROVEAN^c^	Blind test set	78.2	68.7	83.0	75.2	79.0	0.57
SIFT^d^	Blind test set	74.4	62.7	88.0	73.2	76.7	0.53
PolyPhen-2^e^	Blind test set	72.3	60.6	89.5	72.2	75.1	0.50

*Note*: The benchmark is performed on a test set comprising 10 266 human variations (Table 1, 10% of the total number of SAVs) that is blind with respect to our training set. It could be redundant with respect to the training sets of other methods, leading to a possible overestimation of their performances. We also report our performances in cross-validation for comparison. We increased the size of the blind test set up to 20% of the number of SAVs and the E-SNPs&GO MCC score values were negligibly affected (0.5%, data not shown).

a E-SNPs&GO: this article; SNPs&GO (Calabrese *et al.*, 2009).

b MutPred2.0 (Pejaver *et al.*, 2020).

c PROVEAN (Choi *et al.*, 2012).

d SIFT (Ng and Henikoff, 2001).

e PolyPhen-2 (Adzhubei *et al.*, 2010).

Table 3 includes also a comparative benchmark of our method with other state-of-the-art tools, including our SNPs&GO (Calabrese *et al.*, 2009), SIFT (Ng and Henikoff, 2001), PolyPhen-2 (Adzhubei *et al.*, 2010), PROVEAN (Choi *et al.*, 2012) and MutPred2 (Pejaver *et al.*, 2020), one of the most recent and best-performing approaches in the field. Methods are scored adopting our blind test set (Section 2.1), ensuring a fair evaluation of the performance of our method. However, this does not completely exclude the presence of biases in the evaluation of the other tools (with the exception of our SNPs&GO), since variations included in our blind test may be present in the respective training sets, leading to potential overestimation of their performance.

In Table 3, it appears that in this benchmark our method is performing at the state-of-the-art. Among tested approaches, PROVEAN, SIFT and PolyPhen-2, reporting MCCs of 0.57, 0.53 and 0.50, respectively, are scoring lower than our previous SNPs&GO (that achieves an MCC of 0.58). Our E-SNPs&GO and MutPred2, score with significantly higher MCC values of 0.72 and 0.71, respectively. Noticeably the embedding procedure seems to grasp all the properties extracted by an ensemble of different predictors of functional, structural and physicochemical properties, such as the one used by MutPred2 (including over 50 tools). Looking at individual scoring measures, MutPred2 appears more sensitive while our method reports a higher precision.

A detailed ablation study performed to evaluate the effect of the GO terms on the prediction scores (Supplementary Table S4), indicates that the CC sub-ontology slightly outperforms the others.

### 3.3 Prediction of variants of uncertain significance

**Fig. 2.**
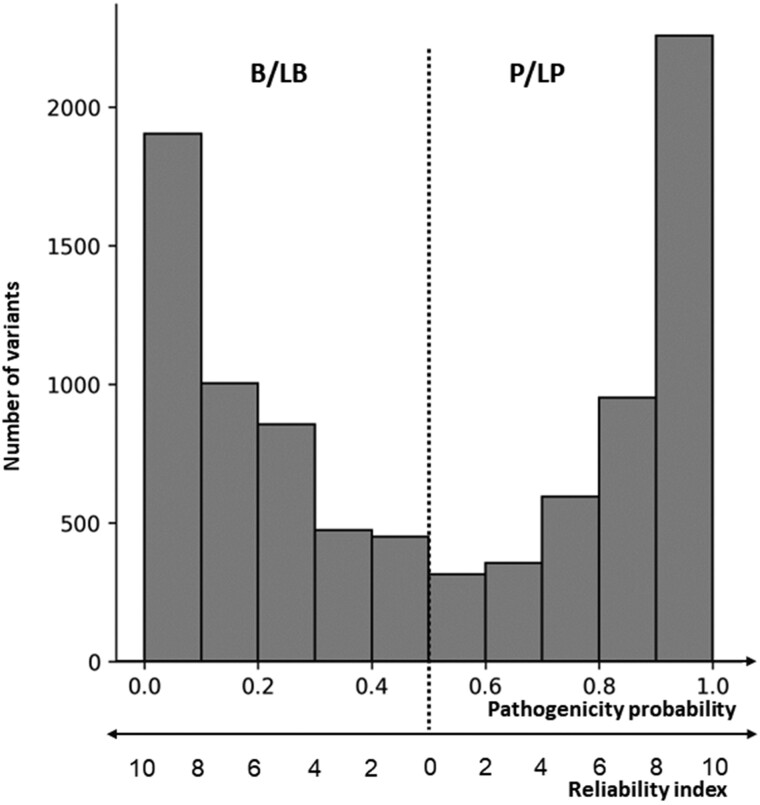
Distribution of predicted pathogenicity probabilities for the dataset of VUS. The value 0.5 discriminates between B/LB and P/LP prediction. Probability values close to either 0 or 1 correspond to prediction with a high reliability index [Equation (1)]

We tested E-SNPs&GO on a dataset of 2588 proteins annotated with 9165 variants of uncertain significance (VUS) extracted from HUMSAVAR (accessed on May 12, 2022). Given that they are uncertain, we cannot assess our performances on this dataset. However, we can sample our predicted annotation in terms of probability and reliability [Equation (6)]. Setting as a reference the probability of being P/LP, Figure 2 shows the distribution of E-SNPs&GO predictions over the whole VUS set as a function of probability and reliability index. A total of 4537 variations are P/LP (pathogenicity probability ≥0.5), while 4628 are B/LB (pathogenicity probability <0.5). The reliability index increases as the probability goes towards 1 or 0 for P/LP and B/LB predictions, respectively [Equation (6)]. In the dataset, 3210 P/LP and 2908 B/LB predictions score with a reliability [RI, Equation (6)] ≥6, accounting for the 67% of VUS. The remaining 33% is predicted with RI lower than 6. For further validation, VUS predictions are available at https://esnpsandgo.biocomp.unibo.it/datasets.

### 3.4 E-SNP&GO web server

E-SNPs&GO web server is available at https://esnpsandgo.biocomp.unibo.it. The server allows users to submit up to 1000 variations per single job. Upon job completion, the results can be visualized on the web page and downloaded in either a tab-separated or a JSON file.

We measured the average E-SNPs&GO runtime by submitting 100 different jobs each including 1000 variations randomly selected from the blind test set. In order to estimate the real execution time for the end user, this experiment was performed in the machine hosting the web server, equipped with one AMD EPYC 7301 CPU with 12 cores, 48 GB of RAM and no GPU available. On average, we obtain a running time of 12.4 ± 4.4 s per variation, when submitting the maximum allowed number of variations per job (1000 variations). This highlights a significant improvement over time-consuming approaches using canonical features such as evolutionary information extracted from multiple sequence alignments.

## 4 Conclusions

We introduce E-SNPs&GO, a method based on language models for annotating whether a single-nucleotide variation is or is not P/LP. We adopt two different protein embedding procedures based on transformers, ESM-1v (Meier *et al.*, 2021) and ProtT5 (Elnaggar *et al.*, 2021). Both embedding methods have been developed and tested on protein variant related problems, such as deep mutational scanning (Marquet *et al.*, 2021; Meier *et al.*, 2021). Here, we address the problem of annotating pathogenic versus benign variations. To this aim, we also add an embedding scheme for functional annotations of wild-type proteins, Anc2Vec (Edera *et al.*, 2022), a method that learns a vector representation for GO terms by preserving ancestor relationships. When benchmarked towards state-of-the-art methods available, E-SNPs&GO well compares to the recently developed MutPred2.0 (Pejaver *et al.*, 2020), which includes as input sequence features derived from some 50 predictors and outperforms previously published methods. Evidently, protein language models learn all the relevant information that can be eventually introduced as input by predictors addressing different tasks.

We prove that embedding models overpass the problem of having as input thousands of different features in order to collect all the relevant features for a reliable annotation of the human pathogenic variations.

## Funding

This work was supported by PRIN 2017 project [2017483NH8 to C.S.] (Italian Ministry of University and Research).

*Conflict of Interest*: none declared.

## Supplementary Material

btac678_Supplementary_Data

## Data Availability

The data underlying this article are available in the article, in its online supplementary material and at the E-SNPs&GO web site: https://esnpsandgo.biocomp.unibo.it.
