# Supplementary material for: E-SNPs&GO: embedding of protein sequence and function improves the annotation of human pathogenic variants
Source: Bioinformatics. 2022 Oct 13;38(23):5168–74. doi: 10.1093/bioinformatics/btac678 (PMC9710551; doi:10.1093/bioinformatics/btac678)
Supplement: btac678_Supplementary_Data [file btac678_supplementary_data.docx]

**Supplementary Material**

**E-SNPs&GO: Embedding of protein sequence and function improves the annotation of hu-man pathogenic variants.**

Matteo Manfredi^1,^°, Castrense Savojardo^1,^°, Pier Luigi Martelli^1,*^ and Rita Casadio^1^

^1^Biocomputing Group, Department of Pharmacy and Biotechnology, Via San Giacomo 9/2, 40126, Bologna, Italy

°Equally contributed.

*To whom correspondence should be addressed.

**Supplementary Table 1**. Composition of the 10 cross-validation (CV) subsets of E-SNPs&GO in terms of number of proteins and of Pathogenic/Likely Pathogenic (P/LP) and Benign/Likely Benign (B/LB) Single Aminoacid Variations (SAV). Data are available at https://esnpsandgo.biocomp.unibo.it/datasets/

| **CV subset** | **# of P/LP SRVs** | **# of B/LB SRVs** | **# of proteins** |
| --- | --- | --- | --- |
| 1 | 4,019 | 6,156 | 1,252 |
| 2 | 3,968 | 6,133 | 1,232 |
| 3 | 3,952 | 6,125 | 1,227 |
| 4 | 4,080 | 6,180 | 1,254 |
| 5 | 3,972 | 6,135 | 1,230 |
| 6 | 3,962 | 6,125 | 1,231 |
| 7 | 3,980 | 6,118 | 1,236 |
| 8 | 3,970 | 6,113 | 1,232 |
| 9 | 3,956 | 6,126 | 1,227 |
| 10 | 3,953 | 6,123 | 1,226 |

**Supplementary Table 2.** Model hyperparameters optimized with grid search.

| Model | Parameter name | Tested values | Optimal value |
| --- | --- | --- | --- |
| PCA | Number of components | 100, 300, 900, 1200, 1800, 2400, 3000, 5208 | 2400 |
| SVM | C (cost parameter) | 10.0, 1.0, 0.1 | 1.0 |
| SVM | class balancing | yes, no | yes |
| RBF kernel | gamma | 1.0, 0.1, 0.01, scale=1 / (n_features * var(X)) | scale |

PCA: Principal Component Analysis

SVM: Support Vector Machine

RBF: Radial Basis Function

**Supplementary Table 3.** Summary table compiled according to DOME (Walsh et al., 2021) recommendations

| **DOME** | Version | 1.0 |
| --- | --- | --- |
| **Data** | Provenance | HUMSAVAR (UniProt Consortium, 2021), accessed on Aug 4th, 2021 and ClinVar (Landrum et al., 2020), accessed on March 29th, 2021. 13,661 protein sequences and 111,412 SAVs total. N_pos_= 43,895 (P/LP SRVs) and N_neg_= 67,517 (B/LB SRVs). Not previously used. |
|  | Dataset splits | N_pos,train_=39,812, N_neg,train_=61,334. N_pos,blind_=4,083, N_neg,blind_=6,183. 39% positives on training set, 40% positives on blind test set. |
|  | Redundancy between data splits | Maximum pairwise sequence identity between training and testing set is 25% on more than 40% alignment coverage. Enforced with MMseqs2 (Steinegger and Söding, 2017) clustering tool. |
|  | Availability of data | Yes, https://esnpsandgo.biocomp.unibo.it/datasets/ |
| **Optimization** | Algorithm | Support Vector Machines |
|  | Meta-predictions | No |
|  | Data encoding | Variant positions encoded with two protein sequence embeddings, namely ESM-1V (Meier et al., 2021) and Prot T5 XL U50 (Elnaggar et al., 2021). Protein global feature encoding GO terms by means of an ontology embedding model, Anc2Vec (Edera et al., 2022) |
|  | Parameters (p) | 47,097 |
|  | Features (f) | 2,400. Feature selection/dimensionality reduction performed by means of a Principal Component Analysis (PCA) of 5,208 original input features. |
|  | Fitting | p is about half N_pos,train_+N_neg,train_=101,146. The risk of over- and under-fitting is very limited. |
|  | Regularization | No. |
|  | Availability of configuration | Yes, hyperparameters in Supplementary Material (Table 2S). |
| **Model** | Interpretability | Black box, as correlation between input and output is masked. |
|  | Output | Binary classification |
|  | Execution time | About 12s for a single variation. |
|  | Availability of software | Webserver: https://esnpsandgo.biocomp.unibo.it/ |
| **Evaluation** | Evaluation method | 10-fold cross-validation and blind test set. |
|  | Performance measures | Accuracy, Precision, Recall, F1-score, MCC, ROC-AUC. |
|  | Comparison | SIFT (Ng and Henikoff, 2001), PolyPhen-2 (Adzhubei et al., 2010), PROVEAN (Choi et al., 2012), SNPs&GO (Calabrese et al., 2009), MutPred2 (Pejaver et al., 2020) |
|  | Confidence | No |
|  | Availability of evaluation | Yes |

**Supplementary Table 4.** E-SNPs&GO performances upon ablation of different GO subontologies.

| Input | Q2* | Precision* | Recall* | F1-Score* | ROC-AUC* | MCC* |
| --- | --- | --- | --- | --- | --- | --- |
| Seq | 83.3 | 90.8 | 64.5 | 75.4 | 80.1 | 0.654 |
| Seq + MF | 84.5 | 90.0 | 68.6 | 77.9 | 81.8 | 0.677 |
| Seq + CC | 85.4 | 90.0 | 71.2 | 79.5 | 83.0 | 0.695 |
| Seq + BP | 85.0 | 89.5 | 70.6 | 79.0 | 82.6 | 0.687 |
| Seq + MF + CC | 86.1 | 88.1 | 75.1 | 81.1 | 84.3 | 0.708 |
| Seq + MF + BP | 85.8 | 88.3 | 74.1 | 80.6 | 83.8 | 0.702 |
| Seq + CC + BP | 86.4 | 87.5 | 76.8 | 81.8 | 84.8 | 0.715 |
| Seq + MF + CC + BP | 86.8 | 85.7 | 80.1 | 82.8 | 85.6 | 0.722 |

Seq: ESM-1v + ProtTrans T5

MF: Molecular Function

CC: Cellular Component

BP: Biological Process

*: For scoring index definition, see Section 2.6 of the main paper

**
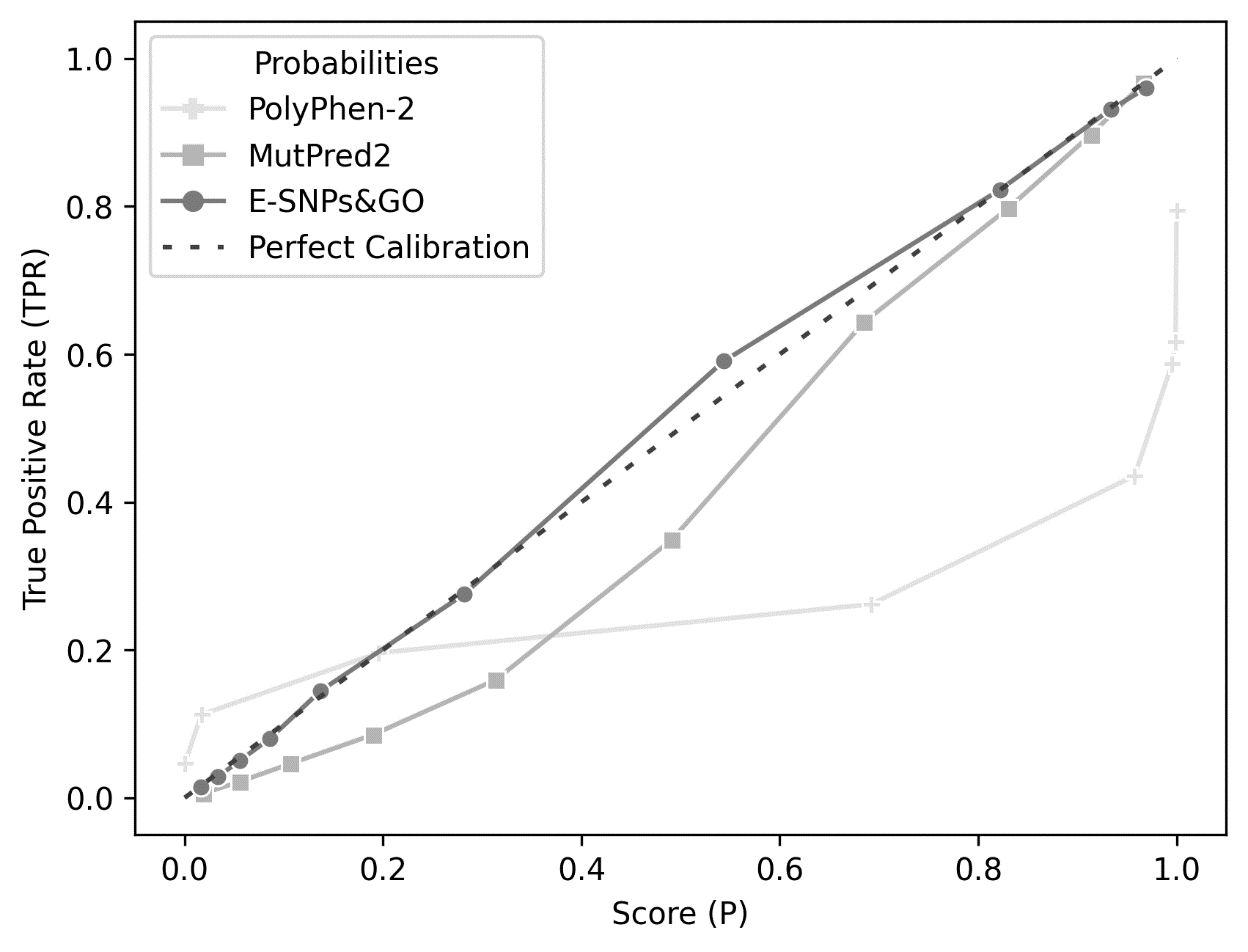
**

**Supplementary Figure 1**. Calibration curves of different methods giving output probabilities on our blind test set. For E-SNPs&GO, the calibration procedure is based on the predictions computed on the training set, and applied to the blind set, preventing information leak.

## References

Adzhubei,I.A. et al. (2010) A method and server for predicting damaging mis-sense mutations. Nat Methods, 7, 248–249.

Calabrese,R. et al. (2009) Functional annotations improve the predictive score of human disease-related mutations in proteins. Hum Mutat, 30, 1237–1244.

Choi,Y. et al. (2012) Predicting the functional effect of amino acid substitutions and indels. PLoS One, 7, e46688.

Edera,A.A. et al. (2022) Anc2vec: embedding gene ontology terms by preserving ancestors relationships. Briefings in Bioinformatics, 23, bbac003.

Elnaggar,A. et al. (2021) ProtTrans: Towards Cracking the Language of Life’s Code Through Self-Supervised Deep Learning and High Performance Computing. arXiv:2007.06225 [cs, stat].

Landrum,M.J. et al. (2018) ClinVar: improving access to variant interpretations and supporting evidence. Nucleic Acids Research, 46, D1062–D1067.

Meier,J. et al. (2021) Language models enable zero-shot prediction of the effects of mutations on protein function. 2021.07.09.450648.

Ng,P.C. and Henikoff,S. (2001) Predicting Deleterious Amino Acid Substitutions. Genome Res, 11, 863–874.

Pejaver,V. et al. (2020). Inferring the molecular and phenotypic impact of amino acid variants with MutPred2. Nat Commun., 11:5918.

Steinegger,M. et al. (2019) Protein-level assembly increases protein sequence recovery from metagenomic samples manyfold. Nat Methods, 16, 603–606.

The UniProt Consortium (2021) UniProt: the universal protein knowledgebase in 2021. Nucleic Acids Research, 49, D480–D489.
